# Characterization of plasmalogen production in facultative anaerobic bacteria and aerobic synthesis in recombinant *Escherichia coli* expressing anaerobic bacterium-derived plasmalogen synthase genes

**DOI:** 10.1128/aem.00940-25

**Published:** 2025-12-22

**Authors:** Rei Irimajiri, Meimi Kuwabara, Yohei Ishibashi, Sakurako Ano, Yasuhiro Fujino, Masanori Honsho, Katsuya Fukami, Shiro Mawatari, Takehiko Fujino, Katsumi Doi

**Affiliations:** 1 Microbial Genetic Division, Institute of Genetic Resources, Faculty of Agriculture, Kyushu University 12923, Fukuoka, Japan; 2 Department of Bioscience and Biotechnology, Graduate School of Bioresource and Bioenvironmental Sciences, Kyushu University 318519, Fukuoka, Japan; 3 Department of Neuroinflammation and Brain Fatigue Science, Graduate School of Medical Sciences, Kyushu University 12923, Fukuoka, Japan; 4 Southern Kyushu and Nansei Islands Innovation Center, Kagoshima University 12851, Kagoshima, Japan; 5 Institute of Rheological Functions of Food, Fukuoka, Japan; Michigan State University, East Lansing, Michigan, USA

**Keywords:** *Lactococcus*, *Enterococcus*, oxygen tolerance, facultative anaerobic bacteria, plasmalogen

## Abstract

Plasmalogens are glycerophospholipids with vital physiological functions, conferring antioxidant properties and contributing to membrane stabilization. While plasmalogen synthase genes *plsA* and *plsR* were identified in the obligate anaerobic bacterium *Clostridium perfringens*, plasmalogen production has not been reported in facultative anaerobes, in which a single gene commonly encodes *plsA*. To establish a cost-effective microbial plasmalogen production system, we screened 38 lactic acid bacterial strains and identified 11 plasmalogen producers, with *Enterococcus faecalis* K-4 exhibiting the highest productivity. Optimization of culture conditions, including the substitution of glucose with lactose and the addition of soy-derived peptides, increased plasmalogen production by 1.5-fold. Heterologous expression of plasmalogen synthesis genes from *E. faecalis* K-4, *Lactococcus cremoris* ATCC BAA-493, *C. perfringens* HN13, and *Bifidobacterium longum* in *Escherichia coli* BL21(DE3) confirmed plasmalogen biosynthesis in all strains. Recombinant *PlsA* from facultative anaerobic *L. cremoris* ATCC BAA-493 exhibited superior oxygen tolerance, enabling high plasmalogen production under aerobic conditions. Structural analysis via liquid chromatography-tandem mass spectrometry revealed consistent plasmalogen species (PE-Pls 16:0/17:0CP, 16:0/19:0CP, and 19:0/17:0CP) across strains and conditions. Plasmalogen-producing *E. coli* recombinants demonstrated enhanced oxidative and osmotic stress resistance, with plasmalogen-expressing cells exhibiting significantly reduced reactive oxygen species accumulation and improved growth in 1.0 M NaCl. Structural modeling using AlphaFold3 indicated that the C-terminal α-helix of *L. cremoris* PlsA contributes to its oxygen tolerance. These findings highlight the potential of facultative anaerobic bacteria, particularly *L. cremoris* ATCC BAA-493, for scalable plasmalogen production and underscore the functional benefits of plasmalogens in enhancing stress resilience.

**IMPORTANCE:**

Plasmalogens are essential glycerophospholipids with crucial physiological functions, including membrane stabilization and antioxidant activity. Recently, supplements that support brain function have gained considerable attention but are expensive due to their extraction from animal tissues and marine sources. In this study, we identified facultative anaerobic bacteria as a cost-effective source for plasmalogen production, offering an accessible strategy to introduce plasmalogens into the diet. Additionally, introducing plasmalogen biosynthetic genes into *Escherichia coli* presents a promising approach for large-scale, efficient plasmalogen production. Notably, for the first time, we achieved aerobic plasmalogen production using recombinant *E. coli* harboring plasmalogen biosynthetic genes from *Lactococcus cremoris*. We hypothesize that the enhanced oxygen tolerance of *L. cremoris* plasmalogen synthase, potentially due to a protective mechanism that prevents oxidative degradation of the [4Fe-4S] cluster, enhances this aerobic production.

## INTRODUCTION

According to the Lipid Maps classification system (https://www.lipidmaps.org/), lipids are classified into eight categories. Among these, glycerophospholipids are the most prevalent components of cell membranes in both eukaryotic cells and bacteria (1, 2). Glycerophospholipids are classified into two types: diacyl and ether. The diacyl type contains acyl bonds at the *sn-*1 and *sn-*2 positions, while the ether type is characterized by an ether bond at the *sn*-1 position, with a phosphate group attached to the *sn*-3 position of the glycerol backbone. Within the ether-type glycerophospholipids, those that feature a vinyl ether bond, where an ether bond and a *cis* double bond are adjacent at the *sn-*1 position, are referred to as plasmalogens (3, 4). Plasmalogens were discovered nearly a century ago, coincidentally, during the staining of animal tissue sections; the plasmalogens were identified as unknown aldehyde-releasing substances in plasma (5). Subsequently, it was determined that plasmalogens are essential lipid components of biological membranes across a wide range of organisms, from microorganisms to animals. In humans, plasmalogens account for approximately 8%–20% of the total phospholipids (6). The plasmalogen content in mammals is tissue-specific, with particularly high concentrations in the brain, heart, skeletal muscle, kidneys, and white blood cells (7). These lipids are associated with several physiological functions, including antioxidant activity, signal transduction, ion transport, cholesterol efflux, and membrane structure formation (7, 8).

In recent years, numerous studies have explored the relationship between various diseases and plasmalogens (7). Decreased plasmalogen levels are strongly associated with Alzheimer’s disease (AD) (9–12). The amyloid hypothesis is currently the most widely accepted explanation for the pathogenesis of AD (13). According to this hypothesis, the amyloid precursor protein is selectively cleaved by β-secretase and γ-secretase, generating toxic amyloid β peptides that accumulate in the brain, leading to neuronal damage and neurodegeneration. In addition, cellular inflammation is triggered by the conversion of oxygen into reactive oxygen species (ROS), which are highly reactive and can damage cellular structures. However, the healthy brain has an innate defense mechanism against oxidative stress, with plasmalogens playing a crucial role in the defense system. Plasmalogens exhibit antioxidant properties and function as sacrificial molecules that protect cells from damage (7). Despite this protective function, plasmalogen levels decrease with age, and it has been confirmed that plasmalogen content in the brains of patients with AD is greatly reduced (14). In response to these findings, various AD therapeutic approaches have been explored (14), with plasmalogen replacement therapy (PRT) emerging as an innovative pharmacological strategy (15). PRT aims to improve brain health by increasing the concentration of plasmalogens and plasmalogen precursors through oral supplementation. The effectiveness of PRT for AD has been demonstrated both *in vitro* and *in vivo* (16, 17), and continued intake of plasmalogens holds promise for the prevention and alleviation of AD. Consequently, the growing interest in plasmalogen supplementation has led to the development of various oral supplements, which require continuous consumption. However, these supplements are associated with challenges due to the high cost of plasmalogens, which are typically derived from animals and marine products. Furthermore, plasmalogen levels in the human brain begin to decrease at the age of 30–40 (18). Thus, there is a need for more cost-effective and sustainable sources of plasmalogens to ensure long-term consumption and AD prevention.

Given that plasmalogens can be efficiently obtained through the mass cultivation of plasmalogen-producing bacteria, we focused on microorganism-derived plasmalogens. In addition, since lipids produced by intestinal bacteria may influence human lipid metabolism (2), we hypothesized that the consumption of plasmalogen-high-producing bacteria and their subsequent colonization in the gut could mitigate the age-related decline in plasmalogen levels.

While plasmalogens are prevalent in obligate anaerobic bacteria and animals, they were previously believed to be absent in aerobic and facultative anaerobic bacteria (19, 20). The presence of plasmalogens in microorganisms was first confirmed in the 1960s (21, 22), with several obligate anaerobic bacteria, including *Clostridium* and *Bifidobacterium* species, identified as plasmalogen producers (23). Initially, it was assumed that the biosynthetic pathways for plasmalogens in microorganisms and animal cells were identical (24). However, when the biosynthetic pathway for animal plasmalogens was elucidated, it was found that molecular oxygen is required for the desaturation of saturated ether lipids (25). Furthermore, metabolic labeling experiments showed that dihydroxyacetone phosphate, a precursor in the animal plasmalogen biosynthesis pathway, is not a precursor in the anaerobic bacterial pathway (24). These findings led to the confirmation of two distinct biosynthetic pathways, one anaerobic and one aerobic. In recent years, genes encoding enzymes involved in plasmalogen biosynthesis, such as PlsA and PlsR, which catalyze the anaerobic introduction of vinyl ether bonds, have been identified in *Clostridium perfringens* (26). In both obligate and facultative anaerobes (which were previously thought not to synthesize plasmalogens), *plsA* is conserved, suggesting that plasmalogen synthesis may also occur in facultative anaerobes. Given that facultative anaerobes do not require strict anaerobic conditions for growth, these bacteria serve as a promising source for large-scale plasmalogen production. However, while the presence of gene homologs has been confirmed in facultative anaerobic bacteria, it remains unclear whether these organisms produce plasmalogens. Therefore, in this study, we aimed to identify facultative anaerobic bacteria capable of plasmalogen production. In addition, we aimed to isolate optimal strains and culture conditions for plasmalogen production.

In addition, we introduced the *plsA* gene into *Escherichia coli*, a widely used and cost-effective host for heterologous gene expression systems, owing to its efficiency and ability to be rapidly cultivated in large quantities. Previous studies have demonstrated that expression of the *plsA* gene from *C. perfringens*, *Megasphaera elsdenii*, and *Streptococcus mutans* in *E. coli* enables plasmalogen production in bacteria that do not normally produce these lipids (26–28). However, these studies did not confirm plasmalogen production under aerobic conditions. In this study, we introduced plasmalogen biosynthesis genes from various obligate and facultative anaerobes into *E. coli* and evaluated both gene expression and plasmalogen production under aerobic or anaerobic conditions.

## RESULTS

### Plasmalogen-producing strains in facultative anaerobic bacteria

Out of the 38 facultative anaerobic *Lactobacillales* strains tested, 11 were identified as plasmalogen producers, based on the detection of aldehydes, confirming the presence of plasmalogens in facultative anaerobic bacteria (Table 1). Among the identified plasmalogen-producing strains, *E. faecalis* K-4 and *L. cremoris* #8-3 were found to be high producers, with aldehyde levels of 0.45 and 0.56 µg per 1 mg of wet cell weight, respectively. Notably, of the five *L. cremoris* strains tested, only the type strain JCM16167^T^ did not produce plasmalogen.

**TABLE 1:** Strains used in the study and their plasmalogen productivity^*a*^

Strains	Aldehyde (μg) per wet cell mass (mg)	Phospholipids (μg) per wet cell mass (mg)	A/P (%)	Plasmalogen productivity	*plsA* homolog	Source or reference
*Aerococcus christensenii* JCM18985^T^				−	No	(29)
*Aerococcus urinae* JCM18986^T^				−	No	NZ_NARE00000000.1
*Aerococcus urinaehominis* JCM18987^T^				−	No	(29)
*Aerococcus viridans* JCM20461^T^				−	No	(29)
*Carnobacterium maltaromaticum* JCM1154^T^				−	IV70_GL001946	(30)
*Carnobacterium mobile* JCM12516T				−	−	JQMR00000000.1
*Clostridium perfringens* NH13				+	CPE1194–CPE1195	(26)
*Enterococcus faecalis* K-4	0.45	1.96	22.96	+	EFK4_10540	(31)
*Enterococcus faecalis* JCM8726^T^	0.07	1.02	6.86	+	IUZ55_000627	NZ_DACBOF010000002.1
*Enterococcus* sp. NB13	0.04	1.56	2.56	+	NA	This study
*Lactiplantibacillus pentosus* NGRI0225				−	NA	(32)
*Lactiplantibacillus plantarum* subsp. *plantarum* JCM 1149^T^				−	−	(32)
*Lactiplantibacillus plantarum* subsp. *plantarum* NGRI 0315				−	NA	(32)
*Lactiplantibacillus plantarum* subsp. *plantarum* NGRI0524				−	NA	(32)
*Lactiplantibacillus plantarum* subsp. *plantarum* NGRI0529				−	NA	(32)
*Lactobacillus rapi* NGRI0130				−	−	(32)
*Lactobacillus* sp. dG3				−	NA	This study
*Lactococcus cremoris* subsp. *cremoris* ATCC 11602	0.02	0.51	3.92	+	NA	ATCC
*Lactococcus cremoris* subsp. *cremoris* ATCC 11603				+	NA	ATCC
*Lactococcus cremoris* subsp. *cremoris* ATCC 14365				+	NA	ATCC
*Lactococcus cremoris* subsp. *cremoris* ATCC BAA-493				+	LACR_2543	(33)
*Lactococcus cremoris* subsp. *cremoris* JCM16167^T^				−	RU89_GL001778	(34)
*Lactococcus* sp. #8-3				+	NA	This study
*Lentilactobacillus buchneri* JCM 1115^T^				−	−	(35)
*Lentilactobacillus buchneri* subsp*. silagei* MGR2-32				−	−	(36)
*Lentilactobacillus kefiri* JCM5818^T^				−	−	BJVK00000000.1
*Lentilactobacillus otakiensis* JCM 15040^T^				−	−	(37)
*Lentilactobacillus parafarraginis* NRIC 0677				−	−	(30)
*Leuconostoc* sp. T21-2				−	NA	This study
*Leuconostoc mesenteroides* subsp. *mesenteroides* JCM 6124^T^				−	−	(33)
*Limosilactobacillus fermentum* JCM 1173^T^				−	−	CP040910.1
*Loigolactobacillus coryniformis* subsp. *torquens* JCM1166^T^				+	FC16_GL000434	CP017697.1
*Pediococcus acidilactici* JCM 8797^T^				−	−	(38)
*Pediococcus pentosaceus* NGRI 0304				−	NA	(32)
*Pediococcus pentosaceus* NGRI 0305				−	NA	(32)
*Streptococcus agalactiae* JCM 5671^T^				−	−	AEQQ00000000.1
*Streptococcus equinus* JCM7879^T^				+	NCTC12969_00935 NCTC12969_00936	UHFK01000003.1
*Streptococcus mutans* JCM5705^T^				+	D820_02030	(39)

^*a*^ A/P, aldehyde content in phospholipids; NA, not analyzed; No, no homolog; +, positive; and −, negative.

### Optimal plasmalogen production conditions for *E. faecalis* K-4

**Fig 1:**
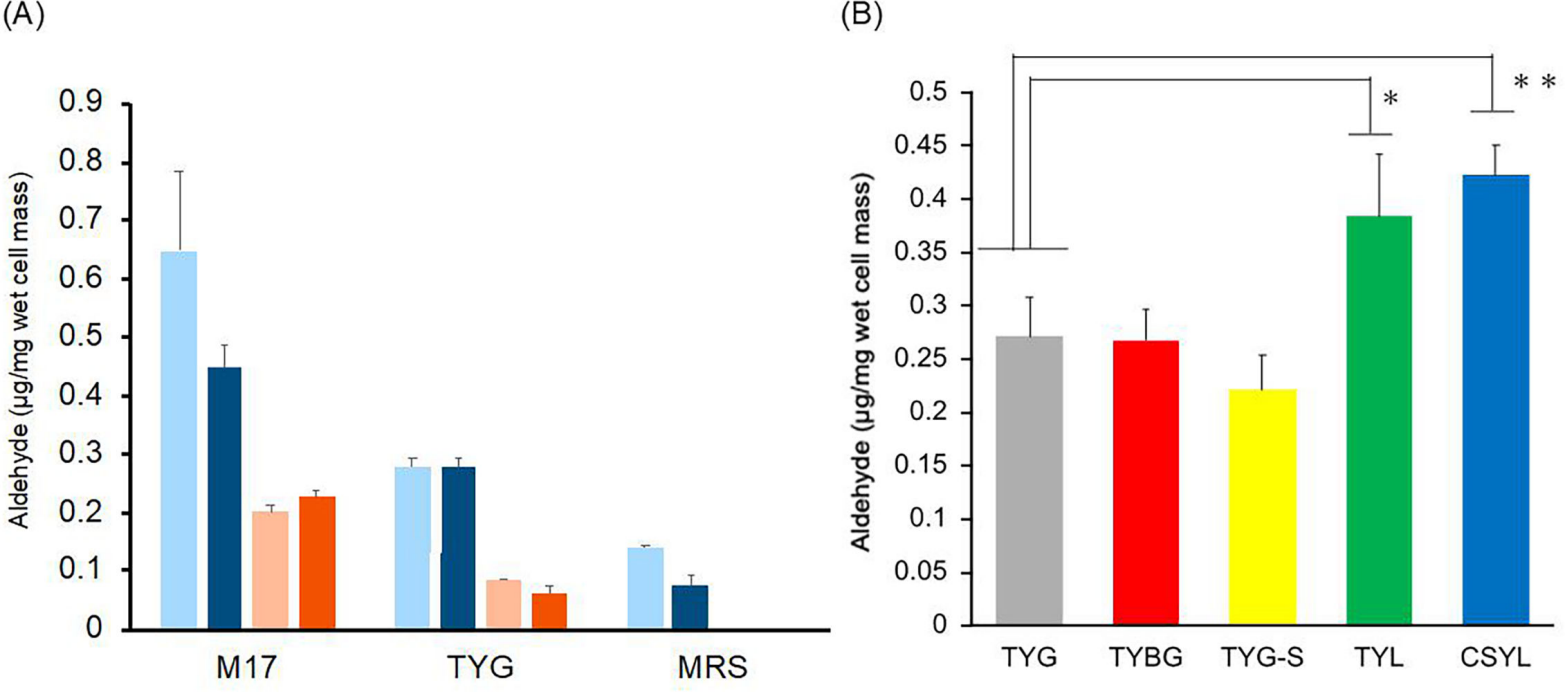
Optimal conditions for plasmalogen production in *E. faecalis* K-4. (**A**) Amount of aldehyde in *E. faecalis* K-4 lipids cultured in different liquid media. Light blue, static culture at 30°C; blue, anaerobic culture at 30°C; salmon, static culture at 45°C; orange, anaerobic culture at 45°C. The measurements were performed three times, and the error bars are shown. (**B**) Amount of aldehyde in *E. faecalis* K-4 lipids cultured in TYG and its modified broth at 30°C. *: *P* < 0.05, **: *P* < 0.01.

When *E. faecalis* K-4 was cultured in M17, MRS, and TYG broths, high levels of aldehydes were detected in K-4 lipids cultured in M17 media, regardless of the culture temperature or the degree of anaerobiosis (Fig. 1A). Notably, the highest aldehyde levels were detected in the K-4 cultured at 30°C in M17 medium. In contrast, K-4 lipids cultured in MRS broth exhibited the lowest aldehyde levels among the three media, and no aldehydes were detected when cultured at 45°C. Furthermore, while no significant difference in aldehyde levels was detected between static and anaerobic cultures, lipids cultured at 45°C contained approximately half the amounts of aldehydes compared to those cultured at 30°C. When comparing the different TYG-based broths, the aldehyde concentration in K-4 lipids was higher in TYL and CSYL broths compared to the control TYG broth (Fig. 1B). Specifically, aldehyde levels increased by 1.4-fold in TYL broth, in which the carbon source was changed from glucose to lactose. In CSYL broth, which contained soybean peptone in TYG broth with lactose as the carbon source, aldehyde concentration increased by 1.5-fold. No significant difference in aldehyde levels was observed in TYBG broth (TYG broth with beef extract). Finally, in TYGS broth, a TYG broth with inorganic salts and ascorbic acid, there was a gradual decrease in the amount of aldehyde.

### Classification of the production plasmalogen species by HPLC peak pattern

**Fig 2:**
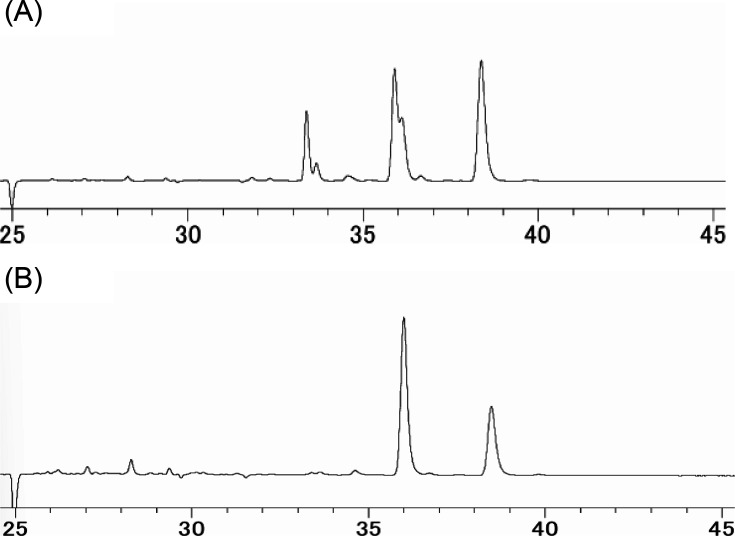
HPLC chromatogram of aldehydes from bacterial cell phospholipids treated with 2,4-dinitrophenylhydrazine-HCl. (**A**) Aldehydes detected in *L. cremoris* ATCC 11602, ATCC 11603, ATCC 14365, and ATCC BAA-493. (**B**) Aldehydes detected in *E. faecalis* K-4, JCM8726^T^, *Enterococcus* sp. NB13, *S. equinus* JCM7879^T^, and *S. mutans* JCM5705^T^.

The extracted lipids were broadly classified into two types: (A) those with two HPLC-detected peaks and (B) those with three HPLC-detected peaks with a retention time of 33 min (Fig. 2). The plasmalogen species produced by *L. cremoris* ATCC 11602, ATCC 11603, ATCC 14365, ATCC BAA-493, and *Lactococcus* sp. #8-3 were classified as type A, and those produced by *E. faecalis* K-4, JCM8726^T^, *Enterococcus* sp. NB13, *S. equinus* JCM7879^T^, and *S. mutans* JCM5705^T^ were classified as type B. Notably, *Loigolactobacillus coryniformis* subsp. *torquens* JCM1166^T^ produced plasmalogens, but the peak patterns did not correspond to any of the peak patterns (data not shown).

### Cloning of the gene for the biosynthesis of plasmalogen

Following the culture tests, we cloned the plasmalogen synthase-encoding gene from *E. faecalis* K-4, a facultative anaerobic bacterium that resulted in a high plasmalogen yield, and from *L. cremoris* ATCC BAA-493, one of the *L. cremoris* strains with a known genome sequence and high productivity, *B. longum* subsp. *suis*, a strict anaerobe known for its ability to produce plasmalogens, and *C. perfringens* HN13, an established plasmalogen producer. Plasmalogen synthase belongs to the 2-hydroxyacyl-CoA dehydratase (HAD) family and is encoded by a two-gene operon, *plsA-plsR*, in *C. perfringens* HN13. Jackson et al. (26) cloned a 4,199-bp genomic fragment containing CPE1194/1195 (*plsA-plsR*) amplified by PCR into the *Nhe*I and *Xho*I sites of pET28a, transformed it into *E. coli* BL21(DE3), and successfully produced plasmalogen. Each ORF has its own start and stop codons, with a short non-coding region between the two genes. Therefore, it is presumed that a *plsA-plsR* mRNA is transcribed by the vector promoter, and the two proteins translated at their respective ribosome binding sites function cooperatively in plasmalogen synthesis. In this study, we cloned the region containing the stop codon and the intergenic region (CPE1194-1195; 4,287 bp) into the pETite N-His SUMO Kan expression vector. In contrast, the corresponding plasmalogen biosynthesis genes in *E. faecalis* K-4, *L. cremoris* ATCC BAA-493, and *B. longum* subsp. *suis* DSM 20211 were all encoded by a single gene. These were named *plsAK4* (4,248 bp), *plsABAA493* (4,311 bp), and *plsAsuis* (4,920 bp), respectively. The cloned genes were inserted into expression vectors, and recombinant *E. coli* BL21(DE3) strains carrying each plasmid were designated as PlsEf, PlsLc, and PlsBs. Additionally, the recombinant *E. coli* strain carrying the plasmid with the *C. perfringens* CPE1194-1195 region was named PlsCp.

### Heterologous expression of PlsA

**Fig 3:**
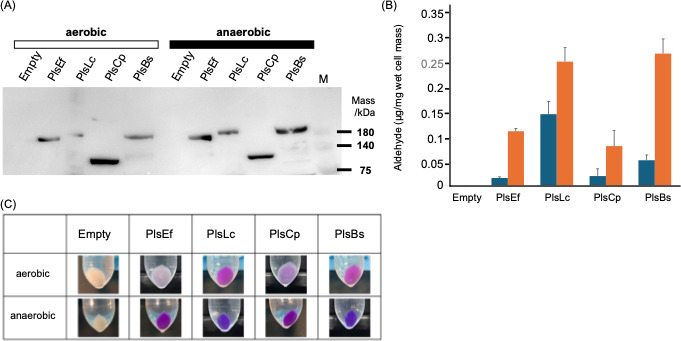
Expression of plasmalogen synthesis genes and production of plasmalogen in recombinant *E. coli*. (**A**) Western Blot analysis of the recombinant PlsA protein. *E. coli* BL21 (DE3) cells transformed with the pETite-Kan vector containing *plsA* or *plsA-plsR* were grown under aerobic or anaerobic conditions and induced with IPTG. For immunodetection, 6′ His-Tag Mouse McAb was used as the primary antibody, and peroxidase-conjugated Affinipure Goat Anti Mouse IgG (H + L) was used as the secondary antibody. M, protein size marker. (**B**) Amount of aldehyde in each recombinant *E. coli* strain in aerobic and anaerobic culture. Navy blue, anaerobic culture; orange, aerobic culture. (**C**) Schiff staining of recombinant *E. coli* cell pellets cultured under aerobic and anaerobic conditions. Empty, *E. coli* BL21 (DE3) harboring the pETite-Kan vector; PlsEf, *E. coli* BL21 (DE3) harboring the *plsA* gene of *E. faecalis* K-4; PlsLc, *E. coli* BL21 (DE3) harboring the *plsA* gene of *L. cremoris* ATCCBAA-493; PlsCp, *E. coli* BL21 (DE3) carrying the *plsA-plsR* genes of *C. perfringens*; and PlsBs, *E. coli* BL21 (DE3) harboring the *plsA* gene of *B. suis*.

Upon induction of each gene expressed in *E. coli* cells, each recombinant PlsA was predicted to have 6×His and SUMO (Small Ubiquitin-like Modifier) tags (approximately 12 kDa) added to its N-terminus, resulting in a protein with an expected molecular weight of approximately 169–190 kDa. To verify the expression of the target proteins, we performed SDS-PAGE and Western blotting (Fig. 3A). Bands corresponding to the expected molecular weights were observed for each recombinant *PlsA*, confirming successful expression. In contrast, no bands were detected at the expected positions in the *E. coli* BL2(DE3) strain transformed with the empty vector. Importantly, all recombinant *E. coli* strains expressed the target protein, irrespective of whether the cultures were grown aerobically or anaerobically. Interestingly, the recombinant *E. coli* strain PlsCp (which expressed the *C. perfringens* gene) showed a band at approximately 128 kDa.

### Plasmalogen production in recombinant *E. coli* expressing PlsA

To evaluate plasmalogen production in the transformed *E. coli* strains expressing PlsA, lipids extracted from each recombinant *E. coli* strain were subjected to HPLC analysis. Both PlsLc and PlsBs produced plasmalogens, as evidenced by the detection of aldehydes in the lipid extracts after acid hydrolysis (Fig. 3B). This was observed under both aerobic and anaerobic cultures. In contrast, PlsEf and PlsCp strains produced plasmalogens only under anaerobic conditions. No plasmalogen production was detected in the *E. coli* BL21(DE3) strain carrying the empty vector (empty strain), serving as a negative control, under any culture condition. Furthermore, plasmalogen production under aerobic conditions was 60%–70% lower in the PlsEf, PlsCp, and PlsBs strains compared to anaerobic conditions. In contrast, the PlsLc strain produced more than three times the amount of plasmalogen as the other strains, even under aerobic conditions.

To further confirm plasmalogen production, Schiff staining was performed. Schiff’s reagent is a colorless liquid containing fuchsin and an excess of sulfurous acid. When aldehydes are present, they react with the reagent, releasing sulfurous acid bound to fuchsin, causing the original magenta color of fuchsin to reappear. Plasmalogens, which contain vinyl ether bonds, undergo acid hydrolysis, breaking the bond and producing aldehydes that react with Schiff reagent to produce a magenta color (26). This was used to determine whether plasmalogens were produced. When the pellets of each recombinant strain were treated with Schiff’s reagent, the empty strain did not exhibit any staining. However, all other recombinant strains stained positively, indicating plasmalogen production. Notably, PlsEf and PlsCp strains, when cultured under aerobic conditions, displayed a paler magenta stain, whereas all the recombinants cultured anaerobically showed more intense magenta staining (Fig. 3C), confirming the presence of plasmalogens in these strains under anaerobic conditions.

### Plasmalogen species and production volume in recombinant PlsA-expressing *E. coli*

**Fig 4:**
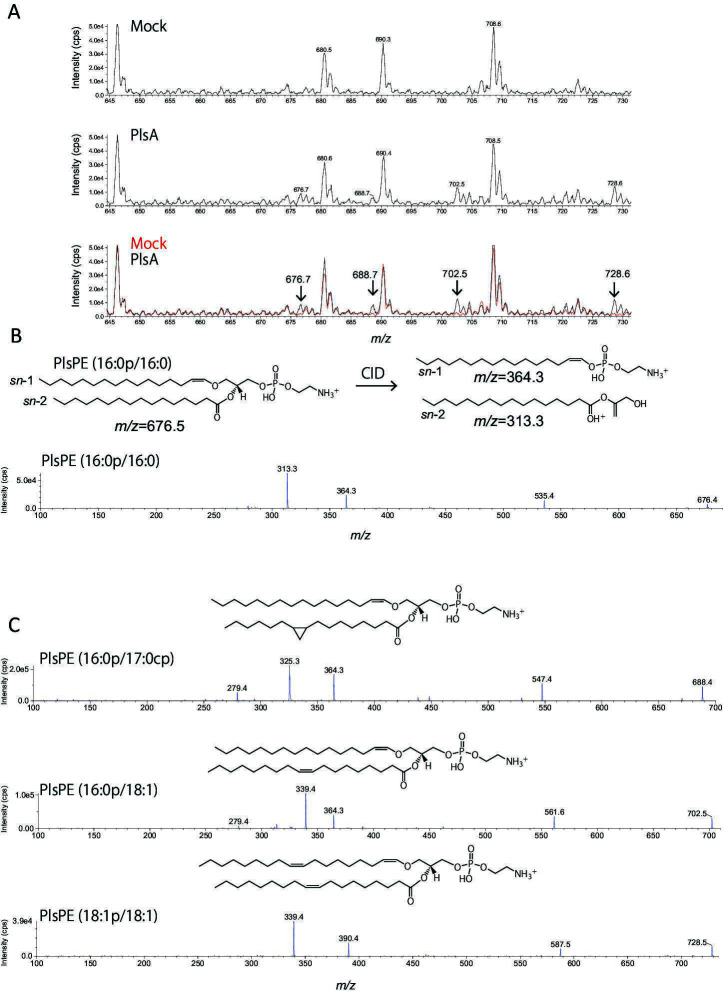
Structural analysis of PlsPE produced in PlsA-expressing *Escherichia coli*. (**A**) Positive ion-mode MS spectra of phospholipid fractions isolated from wild-type and *plsA*-expressing *E. coli* strains. Negative ion signals at *m/z* 676.7, 688.7, 702.5, and 728.6 were exclusively detected in the *plsA*-expressing strain. We measured ions in the *m/z* range of 600–800. In the figure, we extracted and highlighted the region corresponding to the target PlsPE species, ranging from *m/z* 644 to 731. (**B**) Tandem mass spectrometry fragmentation spectrum of the ion at *m/z* 676.5. Structure-specific fragment ions are generated depending on the long-chain alcohol linked via a vinyl ether bond at the *sn*-1 position and an ester-linked fatty acid at the *sn*-2 position. Based on the characteristic fragment ions observed at *m/z* 364.3 and 313.3, the structure of the PlsPE species corresponding to *m/z* 676.5 was identified as 1-(1*Z*)-Hexadecenyl-2-Palmitoyl-*sn*-glycero-3-Phosphoethanolamine (PlsPE 16:0p/16:0). (**C**) Identification of PlsPE species corresponding to *m/z* 688.5, 702.5, and 728.5. Analysis of the MS/MS fragmentation patterns indicated that PlsPE (16:0p/17:0cp), PlsPE (16:0p/18:1), and PlsPE (18:1 p/18:1) were synthesized in PlsA-expressing *E. coli*.

**Fig 5:**
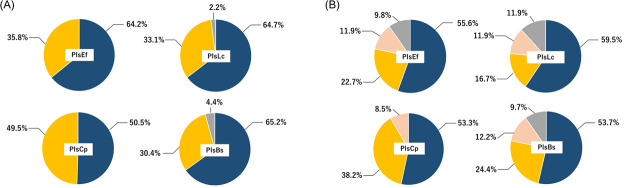
Recombinant *Escherichia coli* producing plasmalogen species and their ratios in (**A**) aerobic culture and (**B**) anaerobic culture. Blue, PE-Pls (16:0/17:0CP); yellow, PE-Pls (16:0/19:0CP); salmon, PE-Pls (19:0/17:0CP); and gray, others.

To investigate the plasmalogen species produced by the recombinant *E. coli* expressing PlsA, MS analysis was performed. Q1 scanning in positive ion mode was used to identify ions specific to the *E. coli* strains expressing PlsA, which were absent in the mock control. Four distinct ions were detected in the PlsA-expressing strain within the LC elution fractions between 19 and 21 min: *m/z* 676.7, 688.7, 702.5, and 728.6 (Fig. 4A). When subjected to collision-induced dissociation (CID) in positive ion mode using ESI, ethanolamine plasmalogen (PlsPE) generates characteristic fragment ions, derived from the long-chain alcohol at *sn*-1 via the vinyl ether bond and ester-linked fatty acid at *sn*-2 (3). For example, the 1-1Z-Hexadecenyl-2-Palmitoyl-sn-glycero-3-Phosphoethanolamine (PlsPE 16:0p/16:0) generates fragment ions at *m/z* 364.3 from sn-1 and *m/z* 313.3 from *sn*-2 upon CID (Fig. 4B). The analysis confirmed that PlsPE was synthesized in the PlsA-expressing strain, as fragment ions at *m/z* 364.3 and 313.3 were observed from the precursor ion *m/z* 676.7 (Fig. 4B). Additionally, a neutral loss of 141 Da from the phosphoethanolamine head was detected at *m/z* 535.4 (Fig. 4B), further supporting the presence of PlsPE (16:0p/16:0) in the recombinant strains. In further MS/MS analysis, fragment ions corresponding to long-chain alcohols and fatty acids at *sn-*1 and *sn*-2, respectively, along with a neutral loss of 141 Da, were detected for *m/z* 688.7, 702.5, and 728.6 (Fig. 4C). Considering that *E. coli* synthesizes cyclopropane-modified fatty acids (2), *m/z* 688.7 was assigned to PlsPE (16:0p/17:0cp), containing cyclopropane-modified palmitoleic acid (17:0cp) at *sn*-2. Similarly, *m/z* 702.5 was identified as PlsPE (16:0p/18:1), and *m/z* 728.6 was identified as 1-(1Z,9Z-Octadecadienyl)−2-oleoyl-sn-glycero-3-phosphoethanolamine (PlsPE [18:1p/18:1]) (Fig. 4C). These findings demonstrate that heterologous expression of PlsA leads to the synthesis of PlsPE, reflecting the endogenous fatty acid composition of *E. coli*. To further investigate the molecular composition of PlsPE, multiple reaction monitoring (MRM) conditions were optimized using fragment ions derived from long-chain alcohols linked to *sn-*1 via vinyl ether linkage as indicators. After optimizing the analytical conditions using standard PlsPE, the relative composition of each PlsPE species was determined. Under aerobic conditions, the main plasmalogen species detected were PlsPE (16:0/17:0CP and 16:0/19:0CP), which accounted for more than 90% of the total plasmalogen production in recombinant *E. coli* (Fig. 5A). Under anaerobic conditions, the three main plasmalogen structures were PlsPE (16:0/17:0CP, 16:0/19:0CP, and 19:0/17:0CP). Together, these plasmalogen species accounted for approximately 90% of the total plasmalogen production in the recombinant strains (Fig. 5B).

### Intracellular reactive oxygen species assay in *E. coli* producing plasmalogens

**Fig 6:**
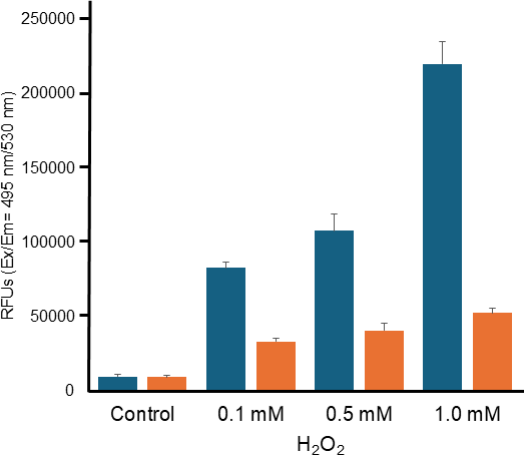
Intracellular ROS assay at each hydrogen peroxide concentration. ROS levels in *E. coli* cells harboring empty vectors (empty strain) and recombinant *E. coli* cells expressing *L. cremoris plsA* (PlsLc) in the absence or presence of H_2_O_2_ treatment. Navy, empty strain; orange: PlsLc.

In the empty strain, a rapid increase in fluorescence was observed as the concentration of hydrogen increased from 0.1 to 1.0 mM (Fig. 6). This suggests that the empty strain exhibited a typical ROS accumulation response, as expected for *E. coli* under oxidative stress conditions. In contrast, in the *E. coli* strain PlsLc, which is a plasmalogen-producing recombinant, the increase in fluorescence was minimal even as the H_2_O_2_ concentration increased. Specifically, when treated with 1.0 mM H_2_O_2_, the fluorescence detected in the PlsLc strain was less than one-quarter of that detected in the empty strain.

### Changes in osmotic pressure tolerance in *E. coli* due to plasmalogen production

**Fig 7:**
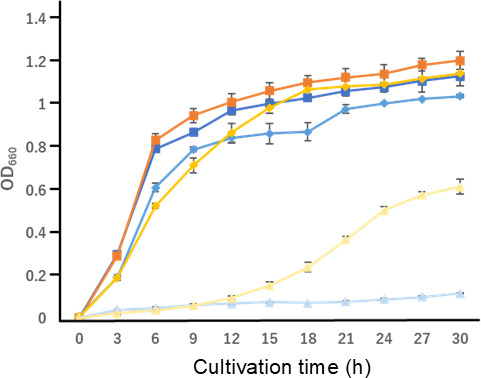
Growth curves of the recombinant and empty strains at different NaCl concentrations. Bacterial growth was monitored by measuring OD_660_ in the cell culture. Dark blue squares, empty strain (NaCl-free); blue diamonds, empty strain (0.5 M NaCl added); light blue triangles, empty strain (1.0 M NaCl added); dark orange squares, strain PlsLc (NaCl-free); orange diamonds, strain PlsLc (0.5 M NaCl added); and yellow triangle, strain PlsLc (1.0 M NaCl added).

When the bacteria were cultured in LB broth containing three different concentrations of NaCl, there was no significant difference between the strains. No significant difference in growth rate was observed between the empty and PlsLc strains when cultured in LB broth containing 0 or 0.5 M NaCl. In both cases, the OD_660_ reached approximately 1.2 after 30 h of culture (Fig. 7), suggesting no significant difference in growth at these NaCl concentrations. However, when cultured in LB broth containing 1.0 M NaCl, the empty strain showed no growth until 30 h of incubation, indicating osmotic pressure sensitivity. In contrast, the PlsLc strain, which produces plasmalogens, showed a significant improvement in osmotic stress tolerance. This strain entered the logarithmic growth phase after 12 h of incubation in the presence of 1.0 M NaCl, and after 30 h of incubation, the OD_660_ reached 0.6.

## DISCUSSION

### Plasmalogen productivity and optimal production conditions for obligate anaerobic bacteria

It has been suggested that plasmalogens are produced not only by obligate anaerobic bacteria, such as *C. perfringens,* but also by facultative anaerobic bacteria such as *E. faecalis* and *S. mutan*s. These findings are particularly significant as they expand the potential of plasmalogen production to lactic acid-fermenting bacteria, which are commonly found in fermented foods. This opens the possibility of regularly ingesting plasmalogens through foods such as fermented dairy products. In the present study, we confirmed the production of plasmalogens in facultative anaerobic bacteria, specifically strains belonging to the Enterococcaceae and Streptococcaceae families. These strains were found to contain genes homologous to the plasmalogen biosynthetic enzymes identified in *C. perfringens* HN13 (PlsA and PlsR), showing sequence identity ranging from 48% to 55% for PlsA and 41% to 42% for PlsR. These existed as a single ORF and encoded regions corresponding to PlsA and PlsR (26). The amino acid sequence similarity above 30% indicates that these homologous genes likely perform similar functions (40), supporting the role of these genes in plasmalogen biosynthesis. However, despite containing genes homologous to those involved in plasmalogen biosynthesis, some strains, such as *C. maltaromaticum* JCM 1154^T^ and *L. cremoirs* subsp. *cremoris* JCM 16167^T^ (41%-51% homologous to the plasmalogen biosynthetic enzymes of *C. perfringens* HN13), did not produce plasmalogens. This highlights the requirement of optimal culture conditions for plasmalogen production, suggesting that some bacterial strains that encode the necessary genes will not produce plasmalogens under all conditions. Thus, to optimize plasmalogen production, it is crucial to understand the impact of various culture conditions on plasmalogen production. Previously, the effect of medium components on plasmalogen production in the luminal bacterial flora has been explored (41); however, the relationship between the medium and plasmalogen productivity in pure cultures remains unknown.

Therefore, we investigated the effects of medium components, oxygen content, and growth temperature on plasmalogen productivity using *E. faecalis* K-4, which is associated with high plasmalogen productivity. The study compared three different media: M17, a complex medium that contains lactose as its carbon source and is suitable for the cultivation of *Lactococcus* species and has a high pH-buffering capacity (42); MRS, most commonly used for the cultivation of lactic acid bacteria (43); and TYG, a simple medium used for the isolation of lactic acid bacteria. Interestingly, M17 led to the highest amount of plasmalogen production, possibly due to the presence of lactose. However, while MRS medium had the highest amount of added sugar, it yielded the lowest amount of aldehyde in K-4 cell lipids. This suggests that simply increasing the sugar content may not be enough to enhance plasmalogen production. Notably, M17 medium, which had the lowest amount of added sugar and lactose, yielded the highest amount of aldehyde. The TYG medium contributed to plasmalogen production; however, the concentrations were lower than those detected in the M17 medium. The presence of lactose and soy peptone in M17 medium likely contributed to the enhanced plasmalogen production. The lactose acts as an excellent carbon source, and soy peptone provides essential carbohydrates and vitamins that further support metabolic processes.

We found that temperature also influenced plasmalogen production. When cultured at 45°C, plasmalogen production was reduced to half of that produced at 30°C. This can be attributed to the preferential allocation of energy toward metabolic pathways that are involved in growth and survival mechanisms at higher temperatures. The biosynthesis of plasmalogens involves the formation of vinyl ether bonds, a process that requires ATP (44). At high temperatures, energy may be diverted to support vital metabolic pathways, reducing the resources available for plasmalogen synthesis. This suggests that lower temperatures are more favorable for plasmalogen production in *E. faecalis* K-4.

Since the production of plasmalogens increased in the M17 medium, the effect of medium components on plasmalogen production was evaluated using this medium. TYL and CSYL were modified to include lactose as the primary carbon source and soy peptone as a nutrient supplement. These modifications led to an increase in plasmalogen production in *E. faecalis* K-4, with TYL medium (lactose only) being particularly effective. The use of soy peptone further contributed to increased plasmalogen production, indicating that it provides additional nutrients that facilitate plasmalogen biosynthesis. CSYL medium also contains a large amount of carbohydrates; thus, it is possible that the amount of plasmalogen produced by *E. faecalis* K-4 is affected by the carbon source in the medium. Compared to the TYG medium, TYBG medium, which contained beef extract, did not significantly impact plasmalogen production, suggesting that the addition of animal-based nutrients does not play a crucial role in enhancing plasmalogen synthesis in this bacterium. The TYGS medium, which contained ascorbic acid and divalent metal ions, resulted in a decrease in plasmalogen production. Ascorbic acid is known to cause the cleavage of the vinyl ether bond in an oxygen-dependent manner (45), and divalent metal ions also act on the hydrolysis of the vinyl ether bond (46). From this, we believe that the addition of metal ions and ascorbic acid, which are abundant in TYG medium, may have promoted the degradation of plasmalogen, decreasing the amount of detected aldehyde in the TYG-S medium. Based on these findings, to maximize plasmalogen production by *E. faecalis* K-4, the following should be prioritized: (i) use of lactose as the carbon source, (ii) supplementation with soy peptone to provide essential nutrients, (iii) avoidance of ascorbic acid, divalent metal ions that may degrade plasmalogens, and (iv) cultivation at lower temperatures, preferably at 30°C, to minimize the diversion of metabolic energy.

### Plasmalogen production under aerobic conditions and the oxygen resistance of PlsA

**Fig 8:**
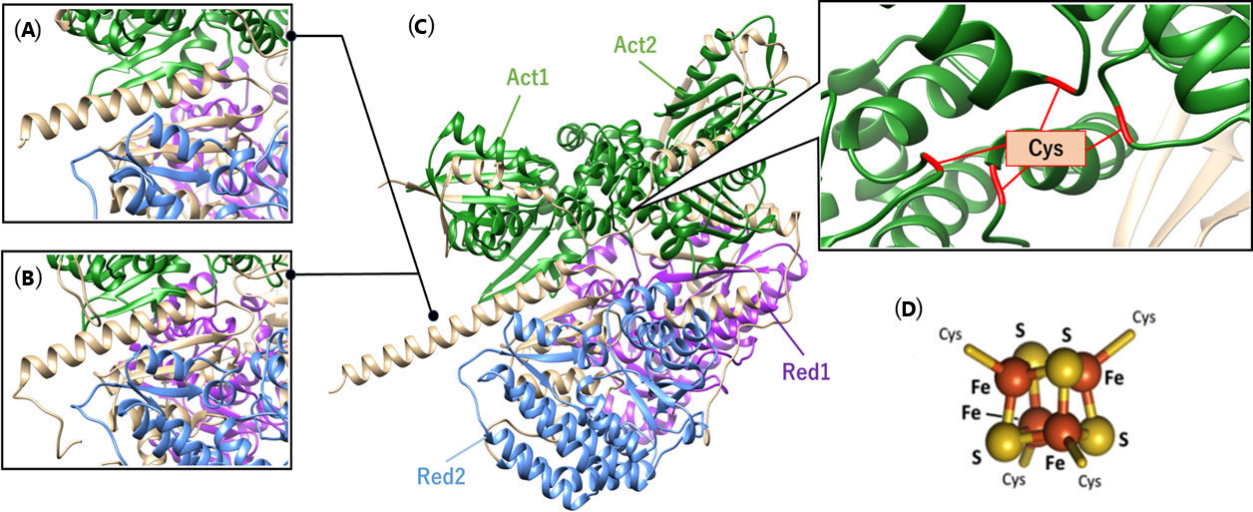
Differences in various plasmalogen synthase enzymes based on AlphaFold3 three-dimensional structure predictions. (**A**) *E. faecalis* K-4, *C. perfringens* NH13, (**B**) *B. suis* DSM20211, and (**C**) *L. cremoris* ATCC BAA-493. Act1 and Act2, activation domains; Red1 and Red2, reduction/dehydration domains. (**D**) [4Fe-4S] cluster.

In this study, we examined plasmalogen production in the genera *Enterococcus*, *Lactococcus*, and *Streptococcus*, which were confirmed to produce plasmalogens. The bacterial strains were classified into two distinct groups based on their aldehyde detection patterns (Fig. 2), reflecting differences in the quantity and types of plasmalogens produced. This classification promoted further investigation into the specificity of the plasmalogen biosynthetic enzyme, as plasmalogens produced varied significantly between strains. This was carried out using *E. coli* as the host organism for heterologous expression of plasmalogen biosynthetic genes, as *E. coli* is not capable of producing plasmalogens (19). Specifically, BL21(DE3), which does not encode *plsA* or *plsR,* was used in this experiment. The empty strain did not produce plasmalogens, as evidenced by no magenta staining during Schiff’s staining, which indicates the absence of aldehyde groups in the cell membranes. In contrast, the recombinant *E. coli* strains that were transformed with *plsA* genes from each plasmalogen-producing bacterium showed magenta staining after Schiff’s reagent treatment, confirming that these strains acquired the ability to produce plasmalogens. Furthermore, in all recombinant strains, the amount of plasmalogen increased under anaerobic conditions compared to aerobic conditions. Among these, the strain PlsLc was suggested to have the ability to produce a relatively large amount of plasmalogen even under aerobic conditions. Next, HPLC analysis revealed that all strains produced plasmalogens; however, PlsLc produced more plasmalogens than the other strains under aerobic conditions. In addition, liquid chromatography-tandem mass spectrometry analysis showed that the plasmalogens produced by all the recombinant strains had the same structure and that the *plsA* gene did not affect the structure of the plasmalogens produced. This is because the plasmalogen synthase encoded by the *plsA* gene introduces a vinyl ether bond, and the structure of the plasmalogen depends on the precursor produced by *E. coli* (23). Based on these findings, we determined that the difference in the amount of plasmalogen produced under aerobic conditions was not due to differences in the structure of the plasmalogen. Therefore, we speculated that the significant decrease in plasmalogen production under aerobic conditions in the strains PlsEf, PlsCp, and PlsBs is due to PlsA activity in the presence of oxygen. PlsA and PlsR in *C. perfringens* are derived from the BCR/HAD family, and it has been reported that proteins belonging to this family are highly oxygen-sensitive (47–49). PlsA, which is also present in the strains PlsEf, PlsCp, and PlsBs, is considered to belong to the BCR/HAD family based on its amino acid sequence. The [4Fe-4S] cluster, commonly found in the BCR/HAD family, is a metal cofactor that plays a role in protein electron transfer. The [4Fe-4S] cluster is easily broken down by oxygen molecules, which is thought to be involved in the decrease in protein activity in the presence of oxygen (50, 51). In PlsA of the strain used in this experiment, cysteine residues are conserved, and since the [4Fe-4S] cluster coordinates with the cysteine residue, it is thought that the [4Fe-4S] cluster also exists in PlsA of the strain. Therefore, it is possible that the oxygen degradation of the [4Fe-4S] cluster contributed to the decrease in PlsA activity. However, since PlsLc produced a large amount of plasmalogen, even under aerobic conditions, PlsA from *L. cremoris* ATCC BAA-493 may have prevented the oxidation of [4Fe-4S] clusters. Therefore, the three-dimensional structure of each PlsA (predicted using AlphaFold3) showed a marked difference in the α-helical structure formed on the C-terminal side. PlsA from *L. cremoris* ATCC BAA-493 has an α-helical structure that is approximately 1.5 times longer than that of other strains, and it is suggested that this structure, which differs from that of other strains, may contribute to the prevention of oxidation of the [4Fe-4S] cluster (Fig. 8). The oxygen resistance of [4Fe-4S] clusters and proteins has been discussed in hydrogenase studies. Hydrogenases are metal enzymes involved in hydrogen metabolism and are found in the intracellular organelles of bacteria and some photosynthetic eukaryotes. There are three types of evolutionarily distinct hydrogenases: Fe, Fe-Fe, and Ni-Fe (52, 53). Fe-type and Fe-Fe-type are irreversibly inactivated in the presence of oxygen and are highly oxygen sensitive. In contrast, Ni-Fe type partly retains activity and is not rapidly inactivated even in the presence of oxygen. Oxygen sensitivity is closely related to the [4Fe-4S] cluster, which exists near the active site. In Ni-Fe hydrogenases, a mechanism that physically excludes oxygen has been proposed as an oxygen-tolerance mechanism. For example, there are reports of mechanisms that prevent oxygen from entering by narrowing the gas channel that leads from the solvent region formed by the protein to the active site and mechanisms that restrict the binding of oxygen to the active site by post-translational modification of the cysteine residues in the active site (54–56). Another mechanism has also been discovered: the active site supplies electrons by forming a [4Fe-3S]-6Cys type cluster rather than a [4Fe-4S]-4Cys type cluster, and the bound oxygen is rapidly decomposed (57). There have also been reports of increased oxygen tolerance by proteins (43, 45, 46). The PlsA from *L. cremoris* BAA-493 used in this study also prevents the oxidative degradation of the [4Fe-4S] cluster by oxygen. This suggests that the PlsLc strain can stably produce plasmalogens even under aerobic conditions. PlsLc is the first strain confirmed to produce plasmalogens under aerobic conditions, and it is expected that large quantities of plasmalogens can be produced in a short time through aerobic cultivation.

### Effects of plasmalogens on recombinant *E. coli* cells

The functional properties of plasmalogens, particularly antioxidant effects, remain largely unexplored, especially those derived from lactic acid bacteria. While plasmalogens from marine sources, such as scallops, have been studied for their potential health benefits, it is unclear whether plasmalogens from lactic acid bacteria or recombinant *E. coli* exhibit similar effects. One of the key challenges of using recombinant *E. coli* to produce plasmalogens as a potential replacement for marine-derived products (e.g., from scallops) is the structural differences in the fatty acid composition. Marine plasmalogens typically contain unsaturated fatty acids at both the *sn*-2 and *sn*-3 positions, while those produced by recombinant *E. coli* may exhibit differences in the fatty acid profile, especially in the *sn*-2 position. Thus, comparing the antioxidant activity of *E. coli*-derived plasmalogens and those from marine products will help determine if recombinant *E. coli* plasmalogens can serve as a viable alternative with similar health benefits. Therefore, we aimed to evaluate the antioxidant potential of plasmalogen produced in recombinant *E. coli* expressing the *plsA* gene from *L. cremoris* (PlsLc), a strain capable of producing plasmalogens under aerobic conditions. Antioxidant activity is an important functional property of plasmalogens, as they are known to protect cells from oxidative stress by scavenging ROS. Previous studies, including those involving *M. elsdenii*-derived plasmalogens expressed in *E. coli* under anaerobic conditions, have demonstrated antioxidant properties through the detection of 2′,7′-dichlorofluorescein diacetate (DCFH-DA) (27). In this study, we expanded on these by investigating plasmalogen production under aerobic conditions using the PlsLc strain and assessing its antioxidant activity.

The results of the intracellular ROS assay demonstrate that plasmalogen production in this strain confers antioxidant properties. This is a significant finding with potential applications in the prevention and treatment of AD. The anti-inflammatory effects of plasmalogen in AD have been confirmed using plasmalogens purified from animal tissues (15). In AD, chronic neuroinflammation exacerbates neuronal damage and accelerates disease progression. Plasmalogens, particularly PlsPE, suppress inflammation by interacting with key inflammatory pathways. This suggests that plasmalogen supplementation could be beneficial in reducing the neuroinflammatory environment that is characteristic of AD. In addition, plasmalogens often have PUFA, such as docosahexaenoic acid (DHA) and eicosapentaenoic acid (EPA), bound to the *sn*-2 position of plasmalogens in humans and animals (58). DHA and EPA exert anti-inflammatory and neuroprotective effects (59). Scallops and sea squirts, which are used as sources of plasmalogens, contain particularly high levels of plasmalogens, combined with DHA and EPA. It is possible that the combined effects of these fatty acids and plasmalogen have inhibitory and ameliorative effects on the onset of AD (60). However, no DHA- (C22:6) or EPA- (C20:5) bound plasmalogens were detected in the PlsLc strain, and it is unclear whether the detected plasmalogens exert the same effects as plasmalogens extracted from scallops and other marine sources, suppressing and improving the onset of AD. Therefore, it is necessary to evaluate the antioxidant effects of plasmalogens derived from PlsLc. In addition to its antioxidant properties, plasmalogen has various other functions and contributes greatly to the stability of the phospholipid bilayer of the cell membrane (2, 7). Plasmalogens have structural and physicochemical properties and are involved in membrane function and interactions between proteins and other components (2, 7). In this study, to evaluate the function of plasmalogens derived from the PlsLc strain, the empty and PlsLc strains were cultured in a high-salt environment. In a high-salt environment, osmotic pressure is increased and cells dehydrate; consequently, biological components, such as proteins and amino acids, become concentrated, making it difficult to maintain cell morphology and ion balance, inhibiting growth (61). In response to this, bacteria regulate ion concentrations, take up water-soluble low-molecular-weight substances, and restore the cell volume (61). As such, the strength of the cell membrane and active substance transport are essential to withstand changes in cell morphology; therefore, it is possible to evaluate the function of plasmalogens derived from the PlsLc strain by observing the growth behavior of the PlsLc strain. *E. coli* is a non-halophilic bacterium, and its growth decreases when the NaCl concentration exceeds 1.0 M when cultured in a rich medium such as LB broth (62) . However, it was confirmed that the PlsLc strain, which harbors the *plsA* gene, is able to grow even in a high-salt environment, and it has acquired osmotic pressure tolerance. This suggests that plasmalogens derived from the PlsLc may have affected the structure of the cell membrane, leading to strengthening of the cell membrane and promotion of material transport. Because the *sn-*1 position of plasmalogens does not contain carbonyl oxygen, intermolecular hydrogen bonds between the head groups are strengthened (23, 63). Furthermore, the parallel arrangement of the proximal regions of the *sn*-1 and *sn*-2 chains, owing to the vinyl ether bond, enables a closer arrangement. Therefore, denser packing of phospholipids in the cell membrane is possible, and membrane rigidity is strengthened (23, 63, 64). In addition, plasmalogens lower the phase transition temperature of the membrane and promote the formation of an inverse hexagonal phase, which is a nonlamellar structure (54). This inverse hexagonal phase is formed when cells are exposed to various environmental stresses that disrupt the bilayer structure of the membrane, such as high osmotic pressure due to high salt environments, high temperatures, and ethanol, as well as functions such as increasing the efficiency of substance uptake and release and promoting ion transport and vesicle transport (64). These functions enable the PlsLc to withstand rapid cell deformation due to osmotic pressure and maintain osmotic pressure regulation. The structural advantages of membranes containing plasmalogens play a crucial role in animals. In particular, improved cell membrane stability and enhanced ion transport are essential for maintaining neurotransmission (63, 65). First, considering cell membrane stability, plasmalogens enable dense packing of phospholipids, improve membrane strength, and ensure appropriate fluidity by lowering the phase-transition temperature of the membrane. This makes it easier for nerve cells to maintain their structure even under oxidative stress and facilitates vesicular transport and membrane fusion at synapses (66). In fact, in synaptosomes isolated from the brains of plasmalogen-deficient mice, the release of neurotransmitters from synaptic vesicles into the presynaptic cleft is inhibited (67). Next, the promotion of ion transport increases the efficiency of the operation of ion channels and transporters due to the reverse hexagonal phase formed by plasmalogens (64). The generation and transmission of action potentials in neurons require a precise flow of ions, including sodium, potassium, and calcium, and the presence of plasmalogen may facilitate the transport of these ions (64). The efficiency of synaptic transmission owing to these functions may promote cognitive function and memory formation (6, 7, 63). PlsLc was able to adapt to a high-salt environment, suggesting that plasmalogens significantly alter the structure and function of cell membranes. This neuroprotective role of plasmalogens is crucial for the maintenance of nerve function and is linked to the structural role in neuronal membranes. Therefore, it is expected that oral intake of plasmalogen derived from PlsLc will maintain and improve the function of nerve cells. We plan to report on this effect elsewhere.

## MATERIALS AND METHODS

### Strains and culture conditions

The strains used in this study are listed in Table 1. *E. faecalis* K-4 was mainly cultured overnight at 30°C in M17 broth (Becton, Dickinson and Company, Sparks, MD, USA). *L. cremoris* ATCC BAA-493 was cultured overnight at 30°C in MRS broth (Becton, Dickinson and Company). *C. perfringens* HN13 was kindly provided by Prof. Jiro Nakayama (Laboratory of Microbial Technology, Faculty of Agriculture, Graduate School, Kyushu University, Japan) and was cultured as previously described (68). Strains obtained from culture collections were cultivated according to the recommended conditions. Other strains were cultivated according to the protocols described in the relevant references. Anaerobic cultures were maintained using AnaeroPak-Anaero (Mitsubishi Gas Chemical, Japan), while aerobic cultures were grown with shaking at 180 rpm.

### Determining the culture conditions for high plasmalogen production

*E. faecalis* K-4 was cultured in various broths, including M17, MRS, TYG, TYL, TYBG, TYGS, and CSYL, at either 30°C or 45°C under aerobic or anaerobic conditions (Tables S1 to S5). Single colonies, obtained by streaking on agar plates, were inoculated into 10 mL of liquid medium to prepare the pre-culture solution. The temperature and oxygen conditions used during the pre-culture were the same as those used for the main culture. The preculture solution was inoculated into fresh liquid broth at a 3% inoculation rate and incubated for 24 h.

### Detection of plasmalogens by HPLC

Bacterial strains were cultured under various conditions, and cells were harvested once sufficient growth was achieved. Bacterial cells were collected and washed thrice with 0.9% sodium chloride solution. The bacterial cell pellets were resuspended in 1 mL of water, followed by 3.75 mL of chloroform/methanol solution (1:2). After the bacterial suspension was sonicated for 10 min, it was incubated at room temperature for 30 min. Following incubation, 1.25 mL of chloroform and 1.25 mL of water were added, and the mixture was centrifuged. The chloroform layer was collected, and 2 mL of chloroform was added to the remaining aqueous layer, followed by mixing and centrifugation. Again, the chloroform layer was collected. The collected chloroform phase was dried under nitrogen gas, and the lipids were resuspended in a hexane/isopropanol (3:2) mixture and filtered through a 0.45 µm filter. The extracted lipids were dried under nitrogen gas, and 0.5 mL of 2,4-dinitrophenylhydrazine (DNPH)-HCl solution (Tokyo Chemical Industry, Tokyo, Japan) was added, mixed, and emulsified by sonication for approximately 10 s. The mixture was then allowed to stand at room temperature for 30 min. Subsequently, 0.5 mL of water was added to the hydrolyzed lipids, mixed, and 1.9 mL of chloroform/methanol (1:2) was added. This mixture was left to stand at room temperature for 10 min before the addition of 0.625 mL of chloroform and 0.625 mL of water. After centrifugation, the chloroform layer was collected, dried under nitrogen gas, resuspended in 100 µL of acetonitrile, and filtered through a 0.45 µm filter. The obtained lipid solution was analyzed using HPLC (An Agilent 1200 series HPLC system; Agilent Technologies Inc., Santa Clara, CA, USA), and the total phospholipids in the bacterial cells were detected using an evaporative light scattering detector (ELSD detector). Next, aldehydes were detected using a UV detector at 356 nm. For total phospholipid detection, a Lichrosphere DIOL column (250 × 3 mm, 5 µm) (Merck KGaA, Darmstadt, Germany) was used at a flow rate of 0.8 mL/min and a column temperature of 50°C. Mobile phase A consisted of hexane, isopropanol, and acetic acid (82:17:1) containing 0.08% triethylamine, while mobile phase B consisted of isopropanol, water, and acetic acid (82:17:1) containing 0.08% triethylamine. For aldehyde detection, an XBridge BEA C18 column (3.0 × 150, 2.5 µm) (Waters, Milford, MA, USA) was used at a flow rate of 0.3 mL/min and a column temperature of 40°C. Mobile phase A was acetonitrile, and mobile phase B was water. The standard aldehyde solutions were prepared as follows: tetradecanal (Tokyo Chemical Industry), hexadecanal (Tokyo Chemical Industry), heptadecanal (Tokyo Chemical Industry), and octadecanal (Tokyo Chemical Industry) were suspended in acetonitrile at a concentration of 1 mg/mL. Each aldehyde solution (200 µL) was dried under nitrogen gas, and DNPH-HCl solution (0.5 mL) was added and mixed. The mixture was emulsified via sonication for approximately 10 s and left to stand at room temperature for 30 min. Next, 0.5 mL of water and 1.9 mL of chloroform/methanol (1:2) were added, the solution mixed, and left to stand at room temperature for 10 min. After adding 0.625 mL of chloroform and 0.625 mL of water, the mixture was centrifuged. The chloroform layer was collected, dried under nitrogen gas, and dissolved in acetonitrile (2 mL). The solution was filtered through a 0.45 µm filter to prepare the aldehyde standard solution. All experiments were conducted in independent triplicate. Strains that exhibited aldehyde production in their bacterial lipids were designated as plasmalogen-producing strains. This method takes advantage of the characteristic properties of plasmalogens containing vinyl ether bonds, one of the three subclasses of glycerophospholipids, which are susceptible to acid hydrolysis and release aldehydes (69).

### Cloning of the *plsA* genes

All primers used in this study are listed in Table S6. PCR was performed using the KOD One PCR Master Mix (TOYOBO, Osaka, Japan) with DNA purified from each bacterial strain as the template. The *plsA* gene from *B. longum* subsp. *suis* DSM 20211 was synthesized by GeneArt Gene Synthesis (Thermo Fisher Scientific, Waltham, MA, USA) based on the nucleotide sequence registered in the database (NZ_JDUC01000003.1). The PCR-amplified *plsA* gene was cloned into the pETite N-His SUMO Kan expression vector using the Expresso T7 SUMO Cloning and Expression System (Lucigen, Middleton, WI, USA). *E. coli* HI-Control 10G (Lucigen) was transformed with the recombinant vector. The resulting transformants were cultured overnight in LB broth (1.0% Bacto Tryptone [Thermo Fisher Scientific], 0.5% Bacto Yeast extract [Thermo Fisher Scientific], and 1.0% NaCl, pH 7.0) containing 30 µg/mL kanamycin at 37°C. Plasmids were subsequently purified using the NucleoSpin Plasmid EasyPure Kit (TaKaRa Bio, Shiga, Japan). The presence of the inserted plasmid fragment was confirmed by nucleotide sequencing. The plasmid containing the *plsA* gene was then transformed into *E. coli* HI-Control BL21(DE3) (Lucigen). *E. coli* BL21(DE3) carrying the empty pETite N-His SUMO Kan plasmid was used as a negative control (named as empty strain).

### Heterologous expression of the *plsA* genes

*E. coli* BL21(DE3) carrying the recombinant plasmids was cultured aerobically or anaerobically at 37°C in 100 mL LB broth containing 30 µg/mL kanamycin. Under aerobic conditions, the culture was shaken at 150 rpm, and 4 h after incubation, induction was conducted under anaerobic conditions with the addition of 0.1 mM IPTG. This was followed by a 10-h incubation in an anaerobic jar using AnaeroPak-Anaero. Following overnight incubation, the cultured bacteria were collected and sonicated using a UD-201 sonicator (TOMY, Tokyo, Japan) at an output power of 3 and a duty cycle of 50. The sonicated bacterial suspension was then centrifuged at 12,000 × *g* and 4°C for 10 min. The supernatant and precipitate were separated using 10% SDS-PAGE. The proteins were transferred to Immobilon-P Transfer Membranes (Merck) via Western blotting. The membrane was incubated with an anti-His-tag antibody (primary antibody: 6′ His-Tag Mouse McAb; Proteintech, Rosemont, IL, USA), followed by detection with a peroxidase-conjugated Affinipure Goat Anti Mouse IgG(H + L) (Proteintech). Membrane-bound peroxidase activity was visualized using the luminescent substrate ImmunoStarZeta (FUJIFILM Wako Pure Chemical, Osaka, Japan).

### Schiff staining

Recombinant *E. coli* BL21(DE3) was cultured in 10 mL LB broth containing 30 µg/mL kanamycin at 37°C, under either aerobic or anaerobic conditions. Expression was induced with 0.1 mM IPTG after 4 h of incubation under aerobic conditions or after 10 h of incubation under anaerobic conditions. After overnight incubation, the bacterial cultures were centrifuged at 12,000 × *g* and 25°C for 1 min to collect the cells. The cell pellet was resuspended in 1 mL of Schiff’s reagent (FUJIFILM Wako Pure Chemical Corp.) and incubated at room temperature for 20 min. Next, the pellet was collected again and visually inspected for color change, indicating the presence of aldehydes in the cells.

### Identification and quantification of plasmalogens from PlsPE by liquid chromatography-electrospray ionization tandem mass spectrometry

*E. coli* BL21(DE3) strains carrying the recombinant plasmids were cultured in 10 mL LB broth containing 30 µg/mL kanamycin at 37°C under aerobic or anaerobic conditions. Expression was induced by the addition of 0.1 mM IPTG after 4 h of incubation under aerobic conditions or after 10 h of incubation under anaerobic conditions. Following overnight incubation, the bacterial cells were collected by centrifugation, and the cell pellet was resuspended in ultrapure water. Both *E. coli* strains expressing *PlsA* and the mock control were subjected to a bead-beating process. The bacterial cell suspension was centrifuged at 15,000 × *g* for 1 min, and the cell pellet was resuspended in 150 µL of distilled water. The cells were then crushed at 3,000 rpm for 60 s using a μT-12 bead beater (TAITEC) with glass beads (diameter: 0.6 mm, AS ONE), followed by incubation on ice for 60 s. This procedure was repeated thrice to prepare the cell lysate. Cellular lipids were extracted from 50 µL of cell lysate by adding 200 µL chloroform/methanol (2:1, vol/vol). After incubation at 37°C for 30 min with shaking at 2,000 rpm using ThermoMixer C (Eppendorf), the mixture was centrifuged at 11,000 × *g* for 3 min. The organic phase containing PlsPE was transferred to autoinjector vials, and cellular lipids were measured using LC-ESI MS/MS (3200 QTRAP, SCIEX, MA, USA). A binary solvent gradient with a 200 µL/min flow rate was used to separate phospholipids and neutral lipids by reverse-phase chromatography using an InertSustain C18 column (2.1 × 150 mm, 5 µm, GL Sciences, Japan) as described in reference 70. The positive ion MS spectra of the lipid fraction from the mock control and *plsA*-expressing *E. coli* were analyzed by a Q1 scan (*m/z* range of 600–800). A product ion scan was performed using collision-induced dissociation to analyze the spectrum observed specifically in *E. coli* expressing *plsA*. Subsequently, multiple reaction monitoring was performed based on the fatty acid composition of *E. coli* (71) (Table S7). For Q3 in MRM, fragment ions derived from the vinyl ether-linked *sn*-1 long-chain alcohol and the head group of PlsPE (Fig. 4) were selected (72). The ionization conditions of PlsPE and the collision energy for generating the target fragment ions were optimized using a 10 µM standard (PlsPE [18:0p/18:1]) (Avanti Polar Lipids, USA).

### Intracellular ROS assay

Intracellular ROS accumulation was measured using 2′,7′-dichlorofluorescein diacetate as a molecular probe. First, recombinant *E. coli* BL21(DE3) was cultured aerobically or anaerobically at 37°C for 16 h, then diluted to OD_600_ = 1.0 and washed thrice with PBS. The pellet was resuspended in 1 mL of DCFH-DA solution. The cells were then treated with 4 µL of 1.0 M H_2_O_2_, while the control sample was treated without H_2_O_2_. The fluorescence derived from DCFH oxidation (produced by intracellular ROS) was measured using a multi-plate reader (Nivo S, Perkin Elmer, Inc., Shelton, CT, USA). The fluorescence was quantified at an excitation wavelength of 488 nm and an emission wavelength of 525 nm. All experiments were independently performed in triplicate.

### Cell proliferation assay under osmotic pressure

Recombinant *E. coli* BL21(DE3) was initially cultured in 100 mL LB broth containing 30 µg/mL kanamycin at 37°C overnight. The diluted culture was then diluted to an OD_600_ of 1.0 in fresh LB broth. The diluted culture was subsequently added to 10 mL of LB broth adjusted to final concentrations of 0.3, 0.5, 0.8, or 1.0 M NaCl. Cultures were grown aerobically or anaerobically. Samples were collected every 3 h, and the OD_600_ was measured using a Multiskan FC microplate reader (Thermo Fisher Scientific). All experiments were performed in independent triplicates.

### Sequence alignment and homology modeling

The reference *plsA* gene and protein sequences were retrieved from the KEGG (https://www.kegg.jp/) and NCBI (https://www.ncbi.nlm.nih.gov/) genome analysis databases, respectively. Homologous sequence alignment was performed using the BLAST tool (https://blast.ncbi.nlm.nih.gov/). The three-dimensional structure of the *plsA* protein was predicted using AlphaFold 3 online program (https://github.com/deepmind/alphafold).

## SUPPLEMENTAL MATERIAL

Tables S1 to S6 aem.00940-25-s0001.pdf Ingredients of media used; primer sequences.

Table S7 aem.00940-25-s0002.pdf MRM conditions for PlsPE measurement.

ASM does not own the copyrights to Supplemental Material that may be linked to, or accessed through, an article. The authors have granted ASM a non-exclusive, world-wide license to publish the Supplemental Material files. Please contact the corresponding author directly for reuse.

## References

[1] Messias MCF, Mecatti GC, Priolli DG, de Oliveira Carvalho P. 2018. Plasmalogen lipids: functional mechanism and their involvement in gastrointestinal cancer. Lipids Health Dis 17:41. doi:10.1186/s12944-018-0685-9 29514688 PMC5842581

[2] Brown EM, Clardy J, Xavier RJ. 2023. Gut microbiome lipid metabolism and its impact on host physiology. Cell Host Microbe 31:173–186. doi:10.1016/j.chom.2023.01.009 36758518 PMC10124142

[3] Dean JM, Lodhi IJ. 2018. Structural and functional roles of ether lipids. Protein Cell 9:196–206. doi:10.1007/s13238-017-0423-5 28523433 PMC5818364

[4] Nagan N, Zoeller RA. 2001. Plasmalogens: biosynthesis and functions. Prog Lipid Res 40:199–229. doi:10.1016/s0163-7827(01)00003-0 11275267

[5] Dorninger F, Werner ER, Berger J, Watschinger K. 2022. Regulation of plasmalogen metabolism and traffic in mammals: The fog begins to lift. Front Cell Dev Biol 10:946393. doi:10.3389/fcell.2022.946393 36120579 PMC9471318

[6] Paul S, Lancaster GI, Meikle PJ. 2019. Plasmalogens: A potential therapeutic target for neurodegenerative and cardiometabolic disease. Prog Lipid Res 74:186–195. doi:10.1016/j.plipres.2019.04.003 30974122

[7] Braverman NE, Moser AB. 2012. Functions of plasmalogen lipids in health and disease. Biochim Biophys Acta 1822:1442–1452. doi:10.1016/j.bbadis.2012.05.008 22627108

[8] Mankidy R, Ahiahonu PW, Ma H, Jayasinghe D, Ritchie SA, Khan MA, Su-Myat KK, Wood PL, Goodenowe DB. 2010. Membrane plasmalogen composition and cellular cholesterol regulation: a structure activity study. Lipids Health Dis 9:62. doi:10.1186/1476-511X-9-62 20546600 PMC2902472

[9] Senanayake V, Goodenowe DB. 2019. Plasmalogen deficiency and neuropathology in Alzheimer’s disease: causation or coincidence? A&D Transl Res & Clin Interv 5:524–532. doi:10.1016/j.trci.2019.08.003 PMC6804645 31650009

[10] Su XQ, Wang J, Sinclair AJ. 2019. Plasmalogens and Alzheimer’s disease: a review. Lipids Health Dis 18:100. doi:10.1186/s12944-019-1044-1 30992016 PMC6466717

[11] Katafuchi T, Ifuku M, Mawatari S, Noda M, Miake K, Sugiyama M, Fujino T. 2012. Effects of plasmalogens on systemic lipopolysaccharide‐induced glial activation and β‐amyloid accumulation in adult mice. Ann N Y Acad Sci 1262:85–92. doi:10.1111/j.1749-6632.2012.06641.x 22823439

[12] Ifuku M, Katafuchi T, Mawatari S, Noda M, Miake K, Sugiyama M, Fujino T. 2012. Anti-inflammatory/anti-amyloidogenic effects of plasmalogens in lipopolysaccharide-induced neuroinflammation in adult mice. J Neuroinflammation 9:197. doi:10.1186/1742-2094-9-197 22889165 PMC3444880

[13] Hardy JA, Higgins GA. 1992. Alzheimer’s disease: the amyloid cascade hypothesis. Science 256:184–185. doi:10.1126/science.1566067 1566067

[14] Ginsberg L, Rafique S, Xuereb JH, Rapoport SI, Gershfeld NL. 1995. Disease and anatomic specificity of ethanolamine plasmalogen deficiency in Alzheimer’s disease brain. Brain Res 698:223–226. doi:10.1016/0006-8993(95)00931-f 8581486

[15] Bozelli JC Jr, Epand RM. 2021. Plasmalogen replacement therapy. Membranes (Basel) 11:838. doi:10.3390/membranes11110838 34832067 PMC8620983

[16] Che H, Zhang L, Ding L, Xie W, Jiang X, Xue C, Zhang T, Wang Y. 2020. EPA-enriched ethanolamine plasmalogen and EPA-enriched phosphatidylethanolamine enhance BDNF/TrkB/CREB signaling and inhibit neuronal apoptosis in vitro and in vivo. Food Funct 11:1729–1739. doi:10.1039/c9fo02323b 32043504

[17] Yamashita S, Hashimoto M, Haque AM, Nakagawa K, Kinoshita M, Shido O, Miyazawa T. 2017. Oral administration of ethanolamine glycerophospholipid containing a high level of plasmalogen improves memory impairment in amyloid β-infused rats. Lipids 52:575–585. doi:10.1007/s11745-017-4260-3 28551706

[18] Rouser G, Yamamoto A. 1968. Curvilinear regression course of human brain lipid composition changes with age. Lipids 3:284–287. doi:10.1007/BF02531202 17805871

[19] Kamio Y, Kanegasaki S, Takahashi H. 1969. Occurrence of plasmalogens in anaerobic bacteria. J Gen Appl Microbiol 15:439–451. doi:10.2323/jgam.15.439

[20] Goldfine H. 2010. The appearance, disappearance and reappearance of plasmalogens in evolution. Prog Lipid Res 49:493–498. doi:10.1016/j.plipres.2010.07.003 20637230

[21] WEGNER GH, FOSTER EM. 1963. Incorporation of isobutyrate and valerate into cellular plasmalogen by Bacteroides succinogenes. J Bacteriol 85:53–61. doi:10.1128/jb.85.1.53-61.1963 13999496 PMC278089

[22] Goldfine H. 1964. Composition of the Aldehydes of Clostridium butyricum plasmalogens: cyclopropane aldehydes. J Biol Chem 239:2130–2134. 14209938

[23] Guan Z, Goldfine H. 2021. Lipid diversity in clostridia. Biochim Biophys Acta Mol Cell Biol Lipids 1866:158966. doi:10.1016/j.bbalip.2021.158966 33974975 PMC8238869

[24] Goldfine H. 2017. The anaerobic biosynthesis of plasmalogens. FEBS Lett 591:2714–2719. doi:10.1002/1873-3468.12714 28617934

[25] Goldfine Howard. 2022. Plasmalogens in bacteria, sixty years on. Front Mol Biosci 9:962757. doi:10.3389/fmolb.2022.962757 36452453 PMC9702350

[26] Jackson DR, Cassilly CD, Plichta DR, Vlamakis H, Liu H, Melville SB, Xavier RJ, Clardy J. 2021. Plasmalogen biosynthesis by anaerobic bacteria: Identification of a two-gene operon responsible for plasmalogen production in Clostridium perfringens. ACS Chem Biol 16:6–13. doi:10.1021/acschembio.0c00673 33350306 PMC7812594

[27] Zhang F, Yang Z, Zhou Y, Wang B, Xie Z, Yu N, Zhao J, Goldfine H, Dai S, Zhang G, Tian B. 2023. Characterization and heterologous expression of plasmalogen synthase MeHAD from Megasphaera elsdenii. Biochim Biophys Acta Mol Cell Biol Lipids 1868:159358. doi:10.1016/j.bbalip.2023.159358 37348645

[28] Mu R, Momeni S, Krieger M, Xie B, Cao X, Merritt J, Wu H. 2024. Plasmalogen, a glycerophospholipid crucial for Streptococcus mutans acid tolerance and colonization. Appl Environ Microbiol 90:e0150023. doi:10.1128/aem.01500-23 38456674 PMC11022534

[29] Carkaci D, Dargis R, Nielsen XC, Skovgaard O, Fuursted K, Christensen JJ. 2016. Complete genome sequences of Aerococcus christensenii CCUG 28831T, Aerococcus sanguinicola CCUG 43001T, Aerococcus urinae CCUG 36881T, Aerococcus urinaeequi CCUG 28094T, Aerococcus urinaehominis CCUG 42038 BT, and Aerococcus viridans CCUG 4311T. Genome Announc 4:e00302-16. doi:10.1128/genomeA.00302-16 27103727 PMC4841142

[30] Sun Z, Harris HMB, McCann A, Guo C, Argimón S, Zhang W, Yang X, Jeffery IB, Cooney JC, Kagawa TF, et al.. 2015. Expanding the biotechnology potential of lactobacilli through comparative genomics of 213 strains and associated genera. Nat Commun 6:8322. doi:10.1038/ncomms9322 26415554 PMC4667430

[31] Kuwabara M, Irimajiri R, Togo S, Fujino Y, Honsho M, Mawatari S, Fujino T, Doi K. 2023. Complete genome sequence of the thermophilic Enterococcus faecalis strain K-4, isolated from a grass silage in Thailand. Microbiol Resour Announc 12:e0081422. doi:10.1128/mra.00814-22 36971556 PMC10112162

[32] Doi K, Nishizaki Y, Kimura H, Kitahara M, Fujino Y, Ohmomo S, Ohshima T, Ogata S. 2013. Identification of thermo tolerant lactic acid bacteria isolated from silage prepared in the hot and humid climate of Southwestern Japan. Springerplus 2:485. doi:10.1186/2193-1801-2-485 24130959 PMC3795204

[33] Makarova K, Slesarev A, Wolf Y, Sorokin A, Mirkin B, Koonin E, Pavlov A, Pavlova N, Karamychev V, Polouchine N, et al.. 2006. Comparative genomics of the lactic acid bacteria. Proc Natl Acad Sci USA 103:15611–15616. doi:10.1073/pnas.0607117103 17030793 PMC1622870

[34] Lambie SC, Altermann E, Leahy SC, Kelly WJ. 2014. Draft genome sequence of Lactococcus lactis subsp. cremoris HPT, the first defined-strain dairy starter culture bacterium. Genome Announc 2:e00107-14. doi:10.1128/genomeA.00107-14 24604643 PMC3945499

[35] Buron-Moles G, Chailyan A, Dolejs I, Forster J, Mikš MH. 2019. Uncovering carbohydrate metabolism through a genotype-phenotype association study of 56 lactic acid bacteria genomes. Appl Microbiol Biotechnol 103:3135–3152. doi:10.1007/s00253-019-09701-6 30830251 PMC6447522

[36] Irimajiri R, Kuwabara M, Togo S, Fujino Y, Honsho M, Mawatari S, Fujino T, Doi K. 2024. Complete genome sequence of Lentilactobacillus buchneri subsp. silagei MGR2-32 isolated from guinea grass silage in Okinawa, Japan. Microbiol Resour Announc 13:e0069523. doi:10.1128/mra.00695-23 38415643 PMC11008148

[37] Doi K, Mori K, Mutaguchi Y, Tashiro K, Fujino Y, Ohmori T, Kuhara S, Ohshima T. 2013. Draft genome sequence of D-branched-chain amino acid producer Lactobacillus otakiensis JCM 15040^T^, isolated from a traditional Japanese pickle. Genome Announc 1:e00546-13. doi:10.1128/genomeA.00546-13 23929467 PMC3738883

[38] Wu L, Ma J. 2019. The Global Catalogue of Microorganisms (GCM) 10K type strain sequencing project: providing services to taxonomists for standard genome sequencing and annotation. Int J Syst Evol Microbiol 69:895–898. doi:10.1099/ijsem.0.003276 30832757

[39] Song L, Wang W, Conrads G, Rheinberg A, Sztajer H, Reck M, Wagner-Döbler I, Zeng AP. 2013. Genetic variability of mutans streptococci revealed by wide whole-genome sequencing. BMC Genomics 14:430. doi:10.1186/1471-2164-14-430 23805886 PMC3751929

[40] Clark WT, Radivojac P. 2011. Analysis of protein function and its prediction from amino acid sequence. Proteins 79:2086–2096. doi:10.1002/prot.23029 21671271

[41] Allison MJ, Bryant MP, Katz I, Keeney M. 1962. Metabolic function of branched-chain volatile fatty acids, growth factors for ruminococci. II. Biosynthesis of higher branched-chain fatty acids and aldehydes. J Bacteriol 83:1084–1093. doi:10.1128/jb.83.5.1084-1093.1962 13860622 PMC279411

[42] Terzaghi BE, Sandine WE. 1975. Improved medium for lactic streptococci and their bacteriophages. Appl Microbiol 29:807–813. doi:10.1128/am.29.6.807-813.1975 16350018 PMC187084

[43] De MAN JC, Rogosa M, Sharpe ME. 1960. A medium for the cultivation of Lactobacilli. J Appl Bacteriol 23:130–135. doi:10.1111/j.1365-2672.1960.tb00188.x

[44] Honsho M, Fujiki Y. 2023. Asymmetric distribution of plasmalogens and their roles-a mini review. Membranes (Basel) 13:764. doi:10.3390/membranes13090764 37755186 PMC10534842

[45] Yavin E, Gatt S. 1972. Oxygen-dependent cleavage of the vinyl-ether linkage of plasmalogens. 2. Identification of the low-molecular-weight active component and the reaction mechanism. Eur J Biochem 25:437–446. doi:10.1111/j.1432-1033.1972.tb01713.x 5065073

[46] Ansell GB, Spanner S. 1965. The magnesium-ion-dependent cleavage of the vinyl ether linkage of brain ethanolamine plasmalogen. Biochem J 94:252–258. doi:10.1042/bj0940252 14342238 PMC1206435

[47] Schweiger G, Dutscho R, Buckel W. 1987. Purification of 2-hydroxyglutaryl-CoA dehydratase from Acidaminococcus fermentans. An iron-sulfur protein. Eur J Biochem 169:441–448. doi:10.1111/j.1432-1033.1987.tb13631.x 3691501

[48] Bornemann S. 2002. Flavoenzymes that catalyse reactions with no net redox change. Nat Prod Rep 19:761–772. doi:10.1039/b108916c 12521268

[49] Buckel W, Kung JW, Boll M. 2014. The benzoyl-coenzyme a reductase and 2-hydroxyacyl-coenzyme a dehydratase radical enzyme family. Chembiochem 15:2188–2194. doi:10.1002/cbic.201402270 25204868

[50] Kiley PJ, Beinert H. 2003. The role of Fe-S proteins in sensing and regulation in bacteria. Curr Opin Microbiol 6:181–185. doi:10.1016/s1369-5274(03)00039-0 12732309

[51] Green J, Crack JC, Thomson AJ, LeBrun NE. 2009. Bacterial sensors of oxygen. Curr Opin Microbiol 12:145–151. doi:10.1016/j.mib.2009.01.008 19246238

[52] Nicolet Y, Cavazza C, Fontecilla-Camps JC. 2002. Fe-only hydrogenases: structure, function and evolution. J Inorg Biochem 91:1–8. doi:10.1016/s0162-0134(02)00392-6 12121756

[53] Meyer J. 2007. [FeFe] hydrogenases and their evolution: a genomic perspective. Cell Mol Life Sci 64:1063–1084. doi:10.1007/s00018-007-6477-4 17353991 PMC11136429

[54] Buhrke T, Lenz O, Krauss N, Friedrich B. 2005. Oxygen tolerance of the H2-sensing [NiFe] hydrogenase from Ralstonia eutropha H16 is based on limited access of oxygen to the active site. J Biol Chem 280:23791–23796. doi:10.1074/jbc.M503260200 15849358

[55] Duché O, Elsen S, Cournac L, Colbeau A. 2005. Enlarging the gas access channel to the active site renders the regulatory hydrogenase HupUV of Rhodobacter capsulatus O2 sensitive without affecting its transductory activity. FEBS J 272:3899–3908. doi:10.1111/j.1742-4658.2005.04806.x 16045760

[56] Marques MC, Coelho R, De Lacey AL, Pereira IAC, Matias PM. 2010. The three-dimensional structure of [NiFeSe] hydrogenase from Desulfovibrio vulgaris Hildenborough: a hydrogenase without a bridging ligand in the active site in its oxidised, “as-isolated” state. J Mol Biol 396:893–907. doi:10.1016/j.jmb.2009.12.013 20026074

[57] Shomura Y, Yoon KS, Nishihara H, Higuchi Y. 2011. Structural basis for a [4Fe-3S] cluster in the oxygen-tolerant membrane-bound [NiFe]-hydrogenase. Nature 479:253–256. doi:10.1038/nature10504 22002607

[58] MacDonald JI, Sprecher H. 1991. Phospholipid fatty acid remodeling in mammalian cells. Biochim Biophys Acta 1084:105–121. doi:10.1016/0005-2760(91)90209-z 1854795

[59] Palacios-Pelaez R, Lukiw WJ, Bazan NG. 2010. Omega-3 essential fatty acids modulate initiation and progression of neurodegenerative disease. Mol Neurobiol 41:367–374. doi:10.1007/s12035-010-8139-z 20467837

[60] Yamashita S, Kanno S, Honjo A, Otoki Y, Nakagawa K, Kinoshita M, Miyazawa T. 2016. Analysis of plasmalogen species in foodstuffs. Lipids 51:199–210. doi:10.1007/s11745-015-4112-y 26732602

[61] Bremer E, Krämer R. 2019. Responses of microorganisms to osmotic stress. Annu Rev Microbiol 73:313–334. doi:10.1146/annurev-micro-020518-115504 31180805

[62] Sasaki H, Iwata E, Oshima A, Ishida A, Nagata S. 2009. Importance of the transcription of proline transporter ProP gene in quick adaptation of Escherichia coli cells under high salinity. Bull. Soc. Sea Water Sci., Jpn 63:338–342.

[63] Vítová M, Palyzová A, Řezanka T. 2021. Plasmalogens - Ubiquitous molecules occurring widely, from anaerobic bacteria to humans. Prog Lipid Res 83:101111. doi:10.1016/j.plipres.2021.101111 34147515

[64] Chen X, Gross RW. 1994. Phospholipid subclass-specific alterations in the kinetics of ion transport across biologic membranes. Biochemistry 33:13769–13774. doi:10.1021/bi00250a030 7947788

[65] Chong K, Almsherqi ZA, Zhuo R, Deng Y. 2021. Plasmalogen-rich foods promote the formation of cubic membranes in amoeba Chaos under stress conditions. FEBS Open Bio 11:2319–2328. doi:10.1002/2211-5463.13241 PMC8329783 34184425

[66] Glaser PE, Gross RW. 1994. Plasmenylethanolamine facilitates rapid membrane fusion: a stopped-flow kinetic investigation correlating the propensity of a major plasma membrane constituent to adopt an HII phase with its ability to promote membrane fusion. Biochemistry 33:5805–5812. doi:10.1021/bi00185a019 8180209

[67] Brodde A, Teigler A, Brugger B, Lehmann WD, Wieland F, Berger J, Just WW. 2012. Impaired neurotransmission in ether lipid-deficient nerve terminals. Hum Mol Genet 21:2713–2724. doi:10.1093/hmg/dds097 22403185 PMC4880048

[68] Adachi K, Ohtani K, Kawano M, Singh RP, Yousuf B, Sonomoto K, Shimizu T, Nakayama J. 2018. Metabolic dependent and independent pH-drop shuts down VirSR quorum sensing in Clostridium perfringens. J Biosci Bioeng 125:525–531. doi:10.1016/j.jbiosc.2017.12.019 29373309

[69] Mawatari S, Hazeyama S, Fujino T. 2016. Measurement of ether phospholipids in human plasma with HPLC-ELSD and LC/ESI-MS after hydrolysis of plasma with phospholipase A1. Lipids 51:997–1006. doi:10.1007/s11745-016-4170-9 27386871 PMC4958133

[70] Ishibashi Y, Goda H, Hamaguchi R, Sakaguchi K, Sekiguchi T, Ishiwata Y, Okita Y, Mochinaga S, Ikeuchi S, Mizobuchi T, Takao Y, Mori K, Tashiro K, Okino N, Honda D, Hayashi M, Ito M. 2021. PUFA synthase-independent DHA synthesis pathway in Parietichytrium sp. and its modification to produce EPA and n-3DPA. Commun Biol 4:1378. doi:10.1038/s42003-021-02857-w 34887503 PMC8660808

[71] Pramanik J, Keasling JD. 1997. Stoichiometric model of Escherichia coli metabolism: incorporation of growth-rate dependent biomass composition and mechanistic energy requirements. Biotechnol Bioeng 56:398–421. doi:10.1002/(SICI)1097-0290(19971120)56:4<398::AID-BIT6>3.0.CO;2-J 18642243

[72] Zemski Berry KA, Murphy RC. 2004. Electrospray ionization tandem mass spectrometry of glycerophosphoethanolamine plasmalogen phospholipids. J Am Soc Mass Spectrom 15:1499–1508. doi:10.1016/j.jasms.2004.07.009 15465363

