# Supplementary material for: Characterization of plasmalogen production in facultative anaerobic bacteria and aerobic synthesis in recombinant Escherichia coli expressing anaerobic bacterium-derived plasmalogen synthase genes
Source: Appl Environ Microbiol. 2025 Dec 22;92(1):e00940-25. doi: 10.1128/aem.00940-25 (PMC12838417; doi:10.1128/aem.00940-25)
Supplement: Tables S1 to S6 — Ingredients of media used; primer sequences. [file aem.00940-25-s0001.pdf]

Supplementary Tables

Table S1 TYG medium

Difco Casein Digest (Thermo Fisher Scientific Inc.)	1%
Bacto Yeast Extract (Thermo Fisher Scientific Inc.)	0.5%
NaCl (FUJIFILM Wako Pure Chemical Corp.)	0.5%
Glucose (FUJIFILM Wako Pure Chemical Corp.)	0.5%

pH adjusted to 7.0. Autoclaved at 121℃, 20 min.

Table S2 TYL medium

Difco Casein Digest (Thermo Fisher Scientific Inc)	1%
Bacto Yeast Extract (Thermo Fisher Scientific Inc)	0.5%
NaCl (FUJIFILM Wako Pure Chemical Corp.)	0.5%
Lactose (FUJIFILM Wako Pure Chemical Corp.)	0.5%

pH adjusted to 7.0. Autoclaved at 121℃, 20 min.

Table S3 TYBG medium

Difco Casein Digest (Thermo Fisher Scientific Inc)	1%
Bacto Yeast Extract (Thermo Fisher Scientific Inc)	0.5%
Gibco Beef Extract Powder (Thermo Fisher Scientific Inc)	0.5%
NaCl (FUJIFILM Wako Pure Chemical Corp.)	0.5%
Glucose (FUJIFILM Wako Pure Chemical Corp.)	0.5%

pH adjusted to 7.0. Autoclaved at 121℃, 20 min.

Table S4 TYGS medium

Difco Casein Digest (Thermo Fisher Scientific Inc)	1%
Bacto Yeast Extract (Thermo Fisher Scientific Inc)	0.5%
Gibco Beef Extract Powder (Thermo Fisher Scientific Inc)	0.5%
L(+)-Ascorbic Acid (FUJIFILM Wako Pure Chemical Corp.)	0.05%
Magnesium Sulfate (FUJIFILM Wako Pure Chemical Corp.)	0.025%
Disodium β-Glycerophosphate Pentahydrate (FUJIFILM Wako Pure Chemical Corp.)	1.9%
Glucose	0.5%

pH adjusted to 7.0. Autoclaved at 121℃, 20 min.

Table S5 CSYL medium

Difco Casein Digest (Thermo Fisher Scientific Inc)	1%
Bacto Yeast Extract (Thermo Fisher Scientific Inc)	0.5%
Gibco Soy Peptone (Thermo Fisher Scientific Inc)	0.5%
NaCl (FUJIFILM Wako Pure Chemical Corp.)	0.5%
Lactose (FUJIFILM Wako Pure Chemical Corp.)	0.5%

pH adjusted to 7.0. Autoclaved at 121℃, 20 min.

Table S6 Primers used in this study

Name	Sequence (5'→3')	Target
BsplsA-f	GAACAGATTGGAGGTATGGTGGATATTGTTACCGATGC	*plsA* in *B. suis* DSM20211
BsplsA-r	GCGGCCGCTCTATTATTAATCTGCGCTGGTAAAAC	
CpplsAR-f	CGCGAACAGATTGGAGGTTTGTATTACAAAATAGGTATTG	*plsA-plsR* operon in *C. perfringens* NH13
CpplsAR-r	GTGGCGGCCGCTCTATTATTAGTTATTTAATATATATTCA	
EfplsA-f	GAACAGATTGGAGGTATGGTTATGAGAGCAGGGAT	*plsA* in *E. faecalis* K-4
EfplsA-r	GCGGCCGCTCTATTATTATCTTTTTACATTTTCTT	
LcplsA-f	CGCGAACAGATTGGAGGTACACAAATTTATAAAACAGGG	*plsA* in *L. cremoris* ATCC BAA-493
LcplsA-r	GTGGCGGCCGCTCTATTATATGCTTGAGCAGTTTCTAAT	
Inv-f	ACCTCCAATCTGTTCGCGGTGAGCC	Inverse PCR for pETite N-His SUMO Kan
Inv-r	TAATAGAGCGGCCGCCACCGCTGAG	

Red text: Sequences identical to pETite N-His SUMO Kan expression vector.
