# Supplementary material for: Characterization of plasmalogen production in facultative anaerobic bacteria and aerobic synthesis in recombinant Escherichia coli expressing anaerobic bacterium-derived plasmalogen synthase genes
Source: Appl Environ Microbiol. 2025 Dec 22;92(1):e00940-25. doi: 10.1128/aem.00940-25 (PMC12838417; doi:10.1128/aem.00940-25)
Supplement: Table S7 — MRM conditions for PlsPE measurement. [file aem.00940-25-s0002.pdf]

Table S7 MRM conditons for the measurment of PlsPE

Product ion	Target compounds	sn-1 (vinyl ether)
336	PE-PL(14:0/14:0)	14:0
	PE-PL(14:0/16:0)	14:0
	PE-PL(14:0/16:1)	14:0
	PE-PL(14:0/17:0CP)	14:0
	PE-PL(14:0/18:0)	14:0
	PE-PL(14:0/18:1)	14:0
	PE-PL(14:0/19:0CP)	14:0
364	PE-PL(16:0/14:0)	16:0
	PE-PL(16:0/16:0)	16:0
	PE-PL(16:0/16:1)	16:0
	PE-PL(16:0/17:0CP)	16:0
	PE-PL(16:0/18:0)	16:0
	PE-PL(16:0/18:1)	16:0
	PE-PL(16:0/19:0CP)	16:0
362	PE-PL(16:1/14:0)	16:1
	PE-PL(16:1/16:0)	16:1
	PE-PL(16:1/16:1)	16:1
	PE-PL(16:1/17:0CP)	16:1
	PE-PL(16:1/18:0)	16:1
	PE-PL(16:1/18:1)	16:1
	PE-PL(16:1/19:0CP)	16:1
376	PE-PL(17:0CP/14:0)	17:0CP
	PE-PL(17:0CP/16:0)	17:0CP
	PE-PL(17:0CP/16:1)	17:0CP
	PE-PL(17:0CP/17:0CP)	17:0CP
	PE-PL(17:0CP/18:0)	17:0CP
	PE-PL(17:0CP/18:1)	17:0CP
	PE-PL(17:0CP/19:0CP)	17:0CP
392	PE-PL(18:0/14:0)	18:0
	PE-PL(18:0/16:0)	18:0
	PE-PL(18:0/16:1)	18:0
	PE-PL(18:0/17:0CP)	18:0
	PE-PL(18:0/18:0)	18:0
	PE-PL(18:0/18:1)	18:0
	PE-PL(18:0/19:0CP)	18:0
	PE-PL(18:1/14:0)	18:1

390	PE-PL(18:1/16:0)	18:1
	PE-PL(18:1/16:1)	18:1
	PE-PL(18:1/17:0CP)	18:1
	PE-PL(18:1/18:0)	18:1
	PE-PL(18:1/18:1)	18:1
	PE-PL(18:1/19:0CP)	18:1
404	PE-PL(19:0CP/14:0)	19:0CP
	PE-PL(19:0CP/16:0)	19:0CP
	PE-PL(19:0CP/16:1)	19:0CP
	PE-PL(19:0CP/17:0CP)	19:0CP
	PE-PL(19:0CP/18:0)	19:0CP
	PE-PL(19:0CP/18:1)	19:0CP
	PE-PL(19:0CP/19:0CP)	19:0CP

sn-2	Q1	Q3
14:0	620.5	336
16:0	648.5	336
16:1	646.5	336
17:0CP	660.5	336
18:0	676.5	336
18:1	674.5	336
19:0CP	688.5	336
14:0	648.5	364
16:0	676.5	364
16:1	674.5	364
17:0CP	688.5	364
18:0	704.5	364
18:1	702.5	364
19:0CP	716.5	364
14:0	646.5	362
16:0	674.5	362
16:1	672.5	362
17:0CP	686.5	362
18:0	702.5	362
18:1	700.5	362
19:0CP	714.5	362
14:0	660.5	376
16:0	688.5	376
16:1	686.5	376
17:0CP	700.5	376
18:0	716.5	376
18:1	714.5	376
19:0CP	728.5	376
14:0	676.5	392
16:0	704.5	392
16:1	702.5	392
17:0CP	716.5	392
18:0	732.5	392
18:1	730.5	392
19:0CP	744.5	392
14:0	674.5	390

16:0	702.5	390
16:1	700.5	390
17:0CP	714.5	390
18:0	730.5	390
18:1	728.5	390
19:0CP	742.5	390
14:0	688.5	404
16:0	716.5	404
16:1	714.5	404
17:0CP	728.5	404
18:0	744.5	404
18:1	742.5	404
19:0CP	756.5	404
